# Phosphoproteomics and Bioinformatics Analyses of Spinal Cord Proteins in Rats with Morphine Tolerance

**DOI:** 10.1371/journal.pone.0083817

**Published:** 2014-01-02

**Authors:** Wen-Jinn Liaw, Cheng-Ming Tsao, Go-Shine Huang, Chin-Chen Wu, Shung-Tai Ho, Jhi-Joung Wang, Yuan-Xiang Tao, Hao-Ai Shui

**Affiliations:** 1 Department of Anesthesiology, Tungs' Taichung MetroHarbor Hospital, Taichung, Taiwan; 2 Department of Anesthesiology, Tri­Service General Hospital, National Defense Medical Center, Taipei, Taiwan; 3 Department of Pharmacology, National Defense Medical Center, Taipei, Taiwan; 4 Department of Anesthesiology, Taipei Veterans General Hospital and National Yang­ Ming University, Taipei, Taiwan; 5 Department of Pharmacology, College of Medicine, Taipei Medical University, Taipei, Taiwan; 6 Departments of Anesthesiology, Medical Research, Chi Mei Medical Center, Tainan, Taiwan; 7 Department of Anesthesiology, Rutgers, The State University of New Jersey, New Jersey Medical School, Newark, New Jersey, United States of America; 8 Graduate Institute of Medical Sciences, National Defense Medical Center, Taipei, Taiwan; University of California, Los Angeles, United States of America

## Abstract

**Introduction:**

Morphine is the most effective pain-relieving drug, but it can cause unwanted side effects. Direct neuraxial administration of morphine to spinal cord not only can provide effective, reliable pain relief but also can prevent the development of supraspinal side effects. However, repeated neuraxial administration of morphine may still lead to morphine tolerance.

**Methods:**

To better understand the mechanism that causes morphine tolerance, we induced tolerance in rats at the spinal cord level by giving them twice-daily injections of morphine (20 µg/10 µL) for 4 days. We confirmed tolerance by measuring paw withdrawal latencies and maximal possible analgesic effect of morphine on day 5. We then carried out phosphoproteomic analysis to investigate the global phosphorylation of spinal proteins associated with morphine tolerance. Finally, pull-down assays were used to identify phosphorylated types and sites of 14-3-3 proteins, and bioinformatics was applied to predict biological networks impacted by the morphine-regulated proteins.

**Results:**

Our proteomics data showed that repeated morphine treatment altered phosphorylation of 10 proteins in the spinal cord. Pull-down assays identified 2 serine/threonine phosphorylated sites in 14-3-3 proteins. Bioinformatics further revealed that morphine impacted on cytoskeletal reorganization, neuroplasticity, protein folding and modulation, signal transduction and biomolecular metabolism.

**Conclusions:**

Repeated morphine administration may affect multiple biological networks by altering protein phosphorylation. These data may provide insight into the mechanism that underlies the development of morphine tolerance.

## Introduction

Morphine is primarily used to treat severe pain caused by acute injuries and chronic diseases. However, systematic administration of morphine can cause many side effects, including impairment of mental and physical functions, psychological dependence, addiction, and tolerance [1], [2]. Since most of the side effects of morphine occur in the supraspinal regions of the central nervous system (CNS), direct neuraxial administration of morphine to act on spinal cord can prevent the supraspinal side effects and provide effective pain relief [2]. However, repeated neuraxial administration of morphine can still lead to tolerance, which is characterized by loss of analgesic effect of the initial effective dose [3]. Understanding the biomolecular changes associated with repeated neuraxial administration of morphine would be helpful for preventing the development of morphine tolerance.

An animal model in which morphine is repeatedly injected into the spinal cord has been used to mimic the direct neuraxial administration of morphine in patients and to study morphine tolerance at the spinal cord level [4], [5]. Morphine tolerance induced by systematic administration is abolished in spinalized animals [6], supporting the premise that morphine tolerance occurs mainly at the spinal cord level rather than at other CNS regions. Thus, the spinal cord is the key target of morphine tolerance. Analysis of molecular events in the spinal cord in an animal model of morphine tolerance would provide a better understanding of the mechanism of this illness [7].

The development of morphine tolerance is thought to be associated with dysregulated phosphorylation of proteins for two reasons. First, morphine exerts its pharmacologic effects by acting at opioid receptors [8], which transduce signals and modulate protein activity via protein phosphorylation. Disturbance of the pharmacologic effects and signal transduction of morphine have been suggested to cause side effects of morphine [9], [10]. Second, studies of cultured cells and brain tissue have shown that morphine and other opioid agonists can affect phosphorylation of certain proteins [11], [12]. We hypothesize that a specific set of phosphoproteins is likely to be involved in the pathogenesis of morphine tolerance. However, the set of phosphoproteins, i.e. the morphine tolerance-related phosphoproteome, has never been explored, especially in the spinal region.

Phosphoproteomics is used to study the phosphorylation of many proteins (the phosphoproteome), rather than individual proteins in a biological sample [13]. Bioinformatics uses computational algorisms to ascertain the physiologic impact of proteins at the systems level [14]. Both approaches can be used to identify proteins whose roles in a disorder have not been established by other traditional methods. To the best of our knowledge, however, these two approaches have never been used to explore the state of phosphoproteins and their biological networks in spinal cord as they relate to development of morphine tolerance. In this study, we used phosphoproteomics and bioinformatics analysis of spinal cord proteins in rats with morphine tolerance to evaluate the impact of morphine on spinal cord in terms of protein phosphorylation, and to better understand the pathophysiologic mechanism that underlies morphine tolerance.

## Materials and Methods

### Animals and drug treatment

All animal experiments were carried out with the approval of the Institutional Animal Care and Use Committee at the National Defense Medical Center, Taiwan, and were consistent with the ethical guidelines of the National Institutes of Health and the International Association for the Study of Pain.

**Figure 1:**
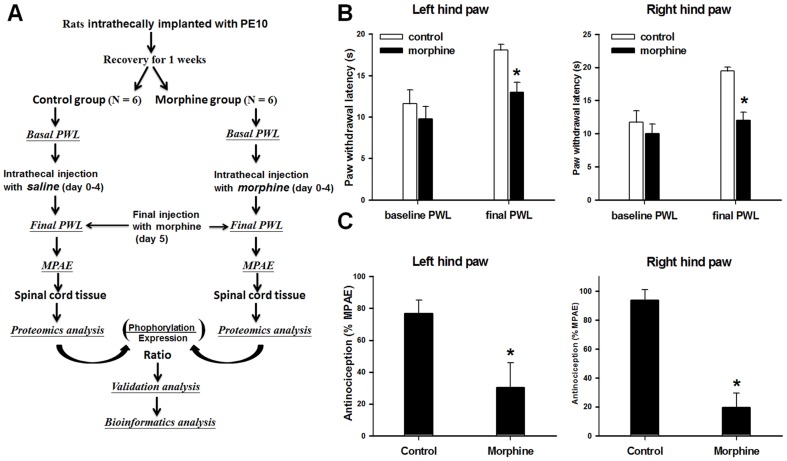
Experimental protocol, nociception, and morphine efficacy. (A) Flow chart of the experimental protocol. (B) Rats were administered twice daily injections of saline (control) or morphine for 4 days. The test morphine injection on day 5 significantly increased the paw withdrawal latency (PWL) of both hind paws in the control group, but not in the morphine-treated group. (C) The calculated maximal possible analgesic effect (MPAE) in both hind paws of morphine-treated rats was significantly lower than that in control rats. *p<0.05 vs. control group.

Male Sprague-Dawley rats (250–300 g) were used in this study. As shown in Fig. 1A, we first implanted a polyethylene-10 catheter into the subarachnoid space of the rats at the rostral level of the spinal cord lumbar enlargement segments as described previously [4], [5], [15]–[17]. The animals were allowed to recover for a week; animals that developed neurologic deficits postoperatively were removed from the study. After recovering from the catheter implantation, the rats were injected intrathecally through the catheter twice daily with saline (10 µL; control group; n = 6) or morphine sulfate (20 µg/10 µL saline, Sigma, St. Louis, MO; n = 6) for 4 consecutive days [5], [18]. On day 5, we injected rats in both groups with morphine sulfate (20 µg/10 µL) to evaluate the analgesic potency [5], [19].

### Nociception tests for morphine efficacy

Nociception tests were carried out at baseline (1 day before the first morphine or saline injection) and on day 5 (30 min after the final injection) as shown in Fig. 1A. Noxious radiant heat (Model 33B Analgesia Meter; IITC Life Science, Woodland Hills, CA, USA) was applied to the plantar surface of each hind paw of the animals. Paw withdrawal latency (PWL) of both hind paws was measured as the time between heat application and paw withdrawal. A cutoff time for the heat application was set to 20 s to prevent tissue damage to the paw [5].

Final and baseline PWLs were compared and used to calculate maximal possible analgesic effect (MPAE) of morphine. MPAE was calculated by the formula: [(final PWL – baseline PWL)/(cutoff time – baseline PWL)] x 100% [5].

### Sample preparation and two-dimensional gel electrophoresis (2-DE)

After nociception testing, the animals were killed and lumbar enlargement segments of spinal cord were harvested. The spinal tissues were immediately lysed and homogenized with a sonication probe in 0.5 mL of lysis buffer (7 M urea, 2 M thiourea, 4% CHAPS, 1% DTT, and 0.5% IPG buffer [pH 3–10]). The lysates were centrifuged at 15000 *g* for 15 min at room temperature to remove insoluble debris. Protein concentration in the supernatant was determined by a modified Bradford method [20].

The spinal proteins (250 µg) from control and morphine-treated groups were loaded onto IPG strips (Immobiline DryStrip 3–10, GE Healthcare, Piscataway, NJ, USA) for simultaneous rehydration. Isoelectric focusing was performed by using a voltage-time program of 50 V for 12 h, 500 V for 1 h, 1000 V for 1 h, and 7000 V to give a total of 140,000 V-h. Immediately after focusing, the IPG strips were equilibrated for 15 min in equilibration buffer (6 M urea, 2% sodium dodecyl sulfate [SDS]; 50 mM Tris [pH 8.4], and 30% glycerol) containing 1% dithiothreitol, and then for 15 min in equilibration buffer containing 2.5% iodoacetamide. The second dimension separation was carried out at 15°C with a vertical electrophoresis system (GE Healthcare) in 1 mm 12.5% acrylamide gels run at 20 mA/gel.

### Gel staining and image analysis

After electrophoresis, the 2-DE gels were subjected to fluorescence staining with Pro-Q Diamond phosphoprotein dye (Invitrogen, Carlsbad, CA, USA) for detection of protein phosphorylation levels [21]. Briefly, the gels were fixed in fixation solution (50% methanol, 10% acetic acid), washed with distilled water twice, and then stained by the Pro-Q Diamond dye for 4 hours. The gels were destained in three successive washes of destaining solution (20% acetonitrile, 50 mM sodium acetate [pH 4]) and three washes of distilled water. Images were obtained by scanning the 2-DE gels with a Typhoon Trio laser scanner (GE Healthcare). For measuring total protein levels, the stained gels were washed with methanol solution (50% methanol, 10% acetic acid), to remove Pro-Q Diamond dye, and were restained with SYPRO Ruby fluorescent dye according to the manufacturer's protocol. Gel images were obtained again by scanning the 2-DE gels with a Typhoon Trio laser scanner.

Spot detection, gel matching, and spot quantification were carried out with 2-DE gel analysis software (ImageMaster 2D platinum, GE Healthcare). To correctly estimate phosphorylation levels, we normalized the phosphorylation intensities of an individual protein revealed by Pro-Q Diamond dye to the total expression level of the same protein revealed by SYPRO Ruby fluorescent dye [21]. The molecular weight (Mr) and isoelectric point (p*I*) of each protein spot were estimated by the software based on the positions of standard markers and standard p*I* positions, respectively.

### In-gel digestion, matrix-assisted laser desorption/ionization-time-of-flight (MADLI-TOF) mass spectroscopy (MS) analysis, and protein identification

Proteins whose phosphorylation level differed significantly between control and morphine-treated groups were excised from the 2-DE gels and digested in the gel with trypsin. The digested proteins were subjected to MALDI-TOF MS analysis (Autoflex II, Bruker Daltonics, Bremen, Germany) to obtain the peptide mass fingerprint (PMF). Each mass spectrum was obtained from the average of signals generated from at least 500 laser shots. The PMFs were processed by using Flexanalysis™ and Biotools™ software (Bruker Daltonics) and were used to search the UniProt database (http://www.uniprot.org/) by using the MS-Fit on-line search engine (http://prospector.ucsf.edu/). For each PMF search to identify a protein, the mass tolerance was set at 150 ppm, and one missed tryptic cleavage was allowed.

### Pull-down assays of phosphopeptide and phosphoprotein

To identify the types and sites of phosphorylation in the most significantly changed protein (i.e. 14-3-3 proteins), we performed pull-down assays at both peptide and protein levels. For pull-down assay of a phophopeptide, the selected protein was in-gel digested into many peptides, and phosphopeptides of the digested protein were precipitated by using Phos-trap™ magnetic beads (Perkin Elmer, San Jose, CA, USA) according to the manufacturer's protocol [22]. MALDI-TOF MS was then used to reveal the precipitated phosphopeptides as well as the original total peptides.

For pull-down assay of phophoproteins, we homogenized spinal cord with RIPA buffer (150 mM NaCl, 0.05% SDS, 1% Triton X-100, 1 mM sodium orthovanadate, 5 mM sodium fluoride, 1x Roche protease inhibitor cocktail, and 50 mM Tris-HCl [pH 7.5]) and precipitated phosphoproteins from the lysate by using a mixed mouse monoclonal anti-phospho-serine and anti-phospho-threonine antibody (Chemicon International, Temecula, CA, USA). Precipitated proteins were then subjected to SDS-polyacrylamide gel electrophoresis (SDS-PAGE) and Western blotting. Western blotting was carried out sequentially by electroblotting proteins onto polyvinylidene difluoride membranes, incubating the membranes with a mouse monoclonal anti-14-3-3 antibody (Chemicon International), and probing with anti-mouse HRP-conjugated secondary antibody. Bands were visualized with an ECL detection kit (Millipore, Billerica, MA, USA). The lysates also were subjected to SDS-PAGE and Western blotting to check the total amount of 14-3-3 protein and glyceraldehyde 3-phosphate dehydrogenase, which served as input controls.

### Bioinformatics analysis

To further estimate the impact of morphine on biological networks, the bioinformatics tool STRING (Search Tool for the Retrieval of Interacting Genes/Proteins) was used to elucidate biological networks regulated by the proteomics-identified phosphoproteins [23]–[25]. The networks were generated by algorithmically assembling the identified phosphoproteins and their interacting proteins from the STRING database. The biological networks were then classified into clusters by protein function [23]–[25].

### Statistics

Student's t-test was used for the statistical comparison of data from control and morphine-treated groups. Differences were considered significant at *p*<0.05. The data are presented as the mean ± SD.

## Results

### Establishment of morphine tolerance in rats

PWLs and MPAEs were measured to ensure the development of morphine tolerance in rats after repeated injections of morphine. As shown in Fig. 1B, the baseline PWLs were similar between the control and morphine-treated rats (*p*>0.05), whereas the final PWLs on day 5 were significantly shortened in morphine-treated rats as compared to those in control rats (*p*<0.05). Because PWL inversely correlates with the extent of pain sensation, the shortened PWLs indicate a reduced analgesic effect of morphine. To ensure the development of morphine tolerance, we also quantified the degree of morphine antinociception by calculating MPAEs. As shown in Fig. 1C, MPAE was significantly less in the morphine-treated rats than in the control rats in both left and right paws (*p*<0.05). Again, this quantitative data confirmed that the analgesic effect of morphine was reduced after repeated morphine injections and verified morphine tolerance.

### Phosphoproteome profiles of spinal cord from control and morphine-treated rats

**Figure 2:**
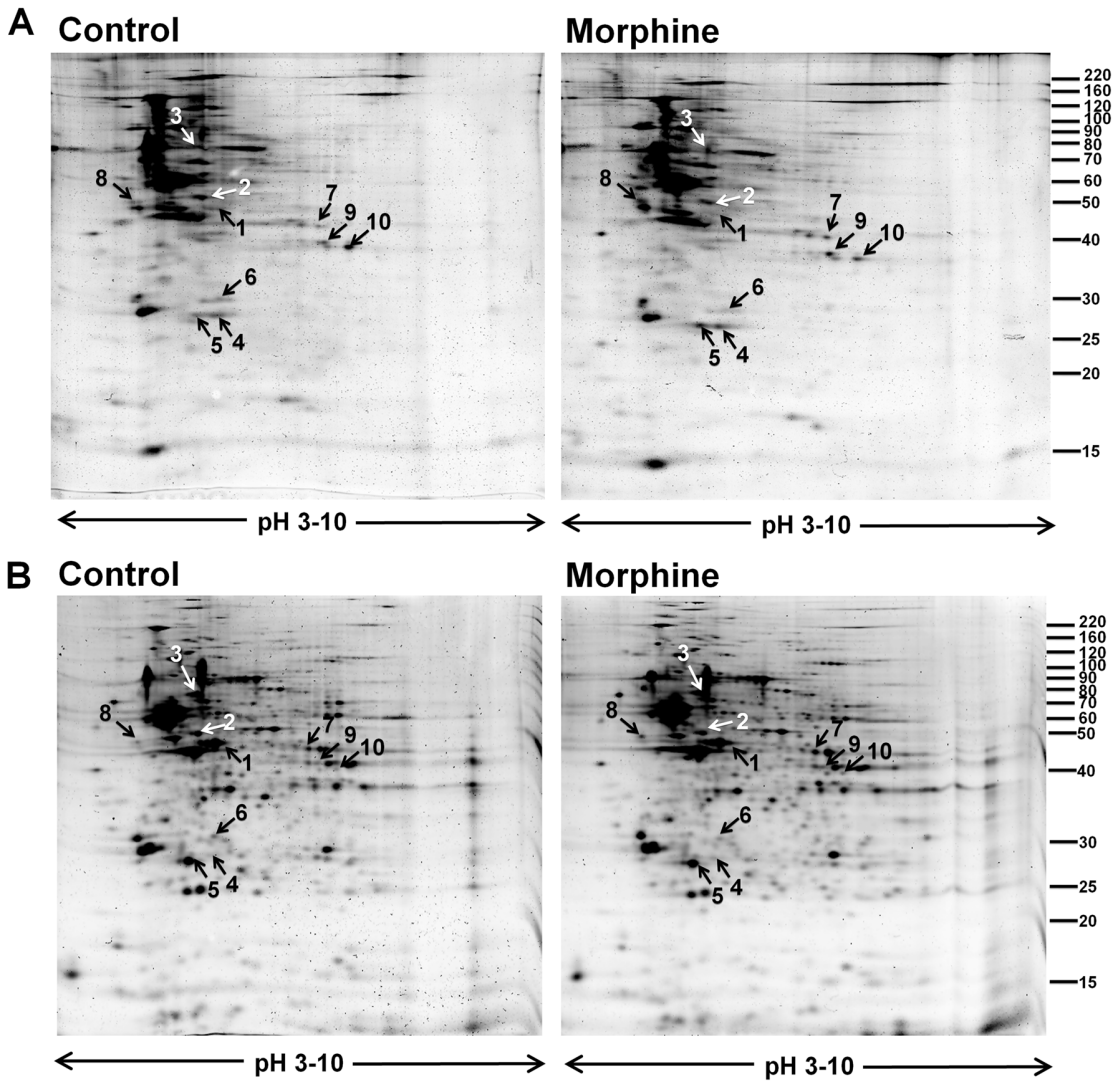
Representative 2-DE gel maps of phosphorylated proteins and total proteins from spinal cord of control and morphine-treated rats. (A) Typical 2-DE gels representing phosphorylated proteins from control (left) and morphine-treated (right) rats; gels were stained with a ProQ Diamond phosphorylation detection dye. (B) The same pair of 2-DE gels from panel A restained with SyproRuby fluorescent dye to show the total protein expression profiles. The protein phosphorylation intensities of the upper gels in panel A were normalized to total protein expression intensities obtained from the lower gels in panel B. The proteins that showed a significant difference in the normalized phosphorylation level are indicated by arrows and labeled with the same numbers used in Table 1. The molecular mass is indicated on the right, and the p*I* range is shown at the bottom of each gel.

Comparative 2-DE-based proteomic analysis revealed a marked difference in phosphorylation pattern of spinal cord proteins between control and morphine-treated rats. Patterns of protein phosphorylation are shown by the representative 2-DE gels stained with ProQ Diamond phosphorylation detection kits (Fig. 2A), and patterns of total protein expression are shown by the same representative 2-DE gels restained with SyproRuby protein staining dye (Fig. 2B). Proteins that exhibited a significant difference in phosphorylation level between control (n = 6) and morphine-treated (n = 6) rats are indicated by arrows. Of those, four proteins were hypophosphorylated and six were hyperphosphorylated in the morphine-treated rats as compared to the control rats.

### Quantification and identification of differentially phosphorylated proteins

**Figure 3:**
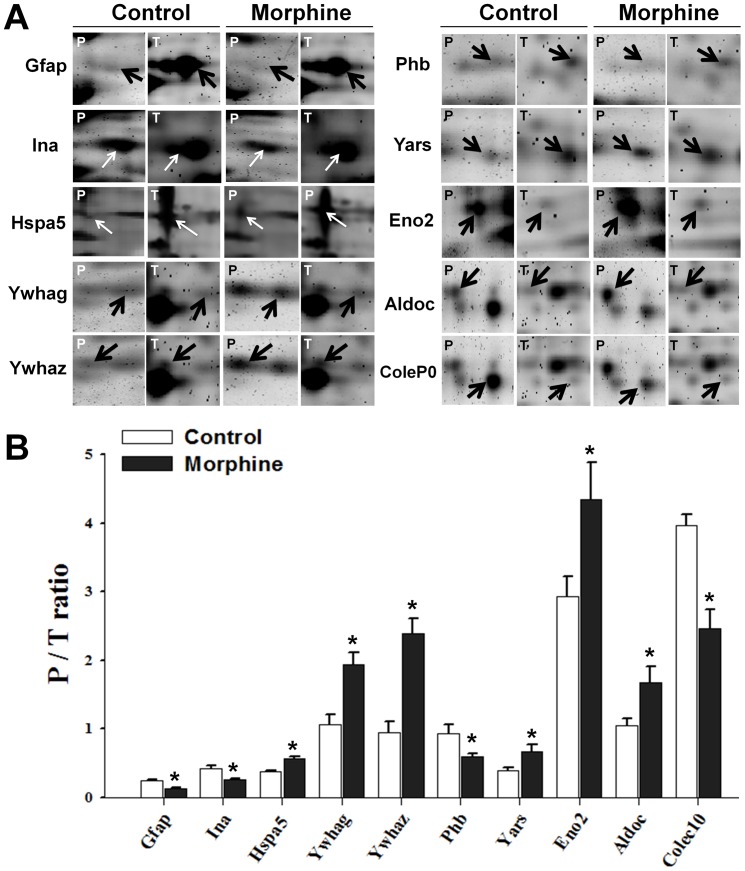
Quantification of differentially phosphorylated proteins. (A) Magnified images of protein spots showing phosphorylation (P) and total amount (T) of the 10 differentially phosphorylated proteins in control and morphine-treated rats. The differentially phosphorylated proteins are indicated by arrows in each magnified image. (B) Statistical and quantification data (P/T ratios) of individual proteins. All differences between the control and morphine-treated groups are significant at the *p*<0.05 level.

The magnified images in Fig. 3A show phosphorylation level (P) and total amount (T) of individual protein spots in control and morphine-treated groups; intensities of protein phosphorylation were divided by corresponding intensities of total protein to give the normalized phosphorylation levels (P/T ratios) shown in Fig. 3B [21]. Differentially phosphorylated proteins were identified by PMF with MALDI-TOF MS, and their various characteristics are shown in Table 1. The proteins included glial fibrillary acidic protein (Gfap), alpha-internexin (Ina), heat shock 70 kDa protein 5 (Hspa5), 14-3-3 protein gamma (Ywhag), 14-3-3 protein zeta/delta (Ywhaz), prohibitin (Phb), tyrosyl-tRNAsynthetase (Yars), gamma-enolase (Eno2), fructose-bisphosphatealdolase C (Aldoc) and collectin sub-family member 10 (Colec10). Phosphorylation levels of Gfap, Ina, Phb, and Colec10 were decreased in the morphine-treated group, whereas phosphorylation levels of the other six proteins were increased.

**Table 1:** Identified proteins.

Spot No.	MOWSE score	Accession No.	Protein name abbreviation	Protein name	Theoretical Mr (kDa)/pI	Sequence coverage (%)
1	16300000	P47819	Gfap	Glial fibrillary acidic protein	49.957/5.4	58.8
2	312000000	P23565	Ina	Alpha-internexin	56.116/5.2	58.8
3	5574	P06761	Hspa5	Heat shock 70 kDa protein 5	72.348/5.1	43.1
4	9645	P61983	Ywhag	14-3-3 protein gamma	28.303/4.8	41.7
5	139899	P63102	Ywhaz	14-3-3 protein zeta/delta	27.771/4.7	51.4
6	39333	P67779	Phb	Prohibitin	29.82/5.6	37.5
7	204	Q4KM49	Yars	Tyrosyl-tRNA synthetase	59.116/6.6	20.1
8	16777	P07323	Eno2	Gamma-enolase	47.141/5.0	27.9
9	1253	P09117	Aldoc	Fructose-bisphosphate aldolase C	39.284/6.7	40.5
10	45.3	D4A7F6	Colec10	Collectin sub-family member 10	30.628/7.5	11.2

The spot number refers to the numbers in Fig. 2. The MOWSE (MOlecular Weight SEarch) score is used to identify proteins from the molecular weight of peptides produced by proteolytic digestion. Mr, theoretical molecular mass; p*I*, isoelectric point. The sequence coverage of matching peptides was calculated by using Biotools™ software.

#### Identification of phosphoproteins involved in neuroplasticity

Gfap (Fig. 2, spot 1; Table 1), a glia cell-specific cytoskeleton protein [26], and Ina (Fig. 2, spot 2, Table 1), a neuron-specific cytoskeleton protein [27], were hypophosphorylated after morphine treatment. Both cytoskeletal proteins play roles in neuroplasticity.

#### Identification of chaperones

Chaperones are involved in maintaining proper conformations of and modulating activities of other proteins. After repeated injection of morphine, phosphorylation levels of one endoplasmic reticulum (ER)-specific chaperone (Hspa5; Fig. 2, spot 3; Table 1) [28] and two 14-3-3 proteins (Fig. 2, spots 4 and 5; Table 1) [29] were increased.

#### Identification of signaling scaffold protein

Scaffold proteins are key regulators of many signaling pathways. Repeated treatment with morphine reduced the phosphorylation level of Phb (Fig. 2, spot 6; Table 1), a scaffold protein that controls the signal transduction of PI3K/Akt, TGF-beta, and Ras/MAPK/ERK [30].

#### Identification of enzymes involved in biomolecular metabolism

The phosphorylation levels of three metabolic enzymes were altered, including two glycolysis enzymes, Eno2 (Fig. 2, spot 8; Table 1) and Aldoc (Fig. 2, spot 9; Table 1) [31], and one protein synthesis-related enzyme Yars (Fig. 2, spot 7; Table 1).

### Identification of types and sites of phosphorylation using pull-down assays

**Figure 4:**
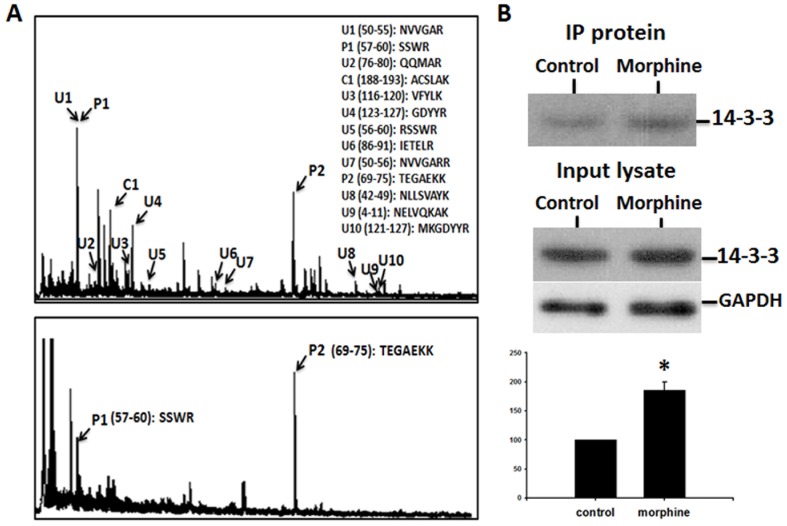
Phosphorylation of 14-3-3 proteins as shown by pull-down assays of phosphopeptide and phosphoprotein. (A) MALDI-TOF MS spectrum of total peptides derived from trypsin digestion of 14-3-3 protein Ywhaz (top panel), and a spectrum of phophopeptides precipitated from total peptides of the digested 14-3-3 protein (bottom panel). The phosphorylated residues represented by the peaks were predicted to be serine and threonine. (B) Upper panel: Western blot showing that more 14-3-3 protein was precipitated by anti-phospho-serine/anti-phospho-threonine antibodies in morphine-treated spinal cord than in control spinal cord. Middle panel: Western blots showing that total 14-3-3 and glyceraldehyde 3-phosphate dehydrogenase (GAPDH) proteins in the lysates of spinal cord were similar in control and morphine-treated rats. Lower panel: Statistical and quantification data of phosphorylation of 14-3-3 proteins.

Pull-down assays were performed to identify the types and sites of phosphorylation in the most significantly changed protein (i.e. 14-3-3 proteins) in 2-DE. Phosphopeptide-enriching magnetic beads induced the precipitation of two major phosphopeptides (P1 and P2 in Fig. 4) from the total peptides of the in-gel digested 14-3-3 protein Ywhaz (spot 5). Based on their amino acid sequences, these peptides were predicted to be serine- and threonine-phosphorylated (Fig. 4A).

Immunoprecipitation of the phosphorylated proteins from total lysates of spinal cord samples and detection by Western blot analysis revealed that 14-3-3 proteins were more abundant in spinal cords from morphine-treated rats than in those from control rats, and confirmed the prediction that 14-3-3 proteins were serine- and threonine-phosphorylated (Fig. 4B).

### Biological networks regulated by the morphine-affected phosphoproteins

**Figure 5:**
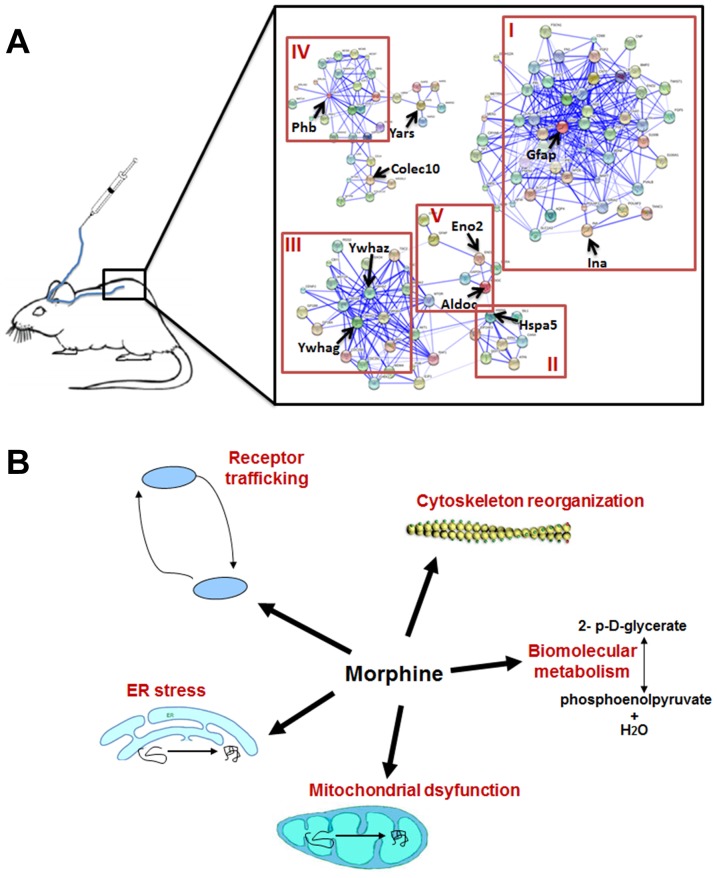
Schematic diagrams of protein-interaction networks and morphine effects. (A) Schematic diagram showing protein-interaction networks affected by the morphine-regulated phosphoproteins. The networks were mapped by using the on-line bioinformatics analysis tool STRING (http://string.embl.de/) and can be classified into five different functional clusters: (I) cytoskeletal reorganization and neuroplasticity, (II) protein folding, (III) protein modulation, (IV) signal transduction, and (V) biomolecular metabolism. (B) Schematic summary showing morphine effects, including receptor trafficking, ER stress, mitochondrial dysfunction, biomolecular metabolism and cytoskeleton reorganization.

Bioinformatics analysis was used to estimate the biological networks impacted by the 10 proteomics-identified phosphoproteins. The biological network clusters, shown in Fig. 5, consisted of cytoskeleton reorganization and neuroplasticity (cluster I), protein folding (cluster II), protein modulation (cluster III), signal transduction (cluster IV), and biomolecular metabolism (cluster V).

## Discussion

To date, phosphoproteomics has not been used to analyze phosphorylation of spinal cord proteins after development of morphine tolerance. Our proteomics and bioinformatics data revealed that repeated intrathecal morphine injections dysregulated the phosphorylation of 10 proteins in rat spinal cord, impacting biological networks associated with various physiologic functions of the CNS. Our results are novel in that the identified phosphoproteins have not been reported previously to be associated with morphine tolerance.

The nervous system requires a degree of plasticity to respond to external stimuli and insults. This plasticity is achieved through cytoskeletal reorganization, which is controlled by phosphorylation of cytoskeletal proteins [32]–[34]. Our study showed that phosphorylation states of two cytoskeletal proteins in the spinal cord, i.e. Gfap and Ina, were altered by repeated administration of morphine. GFAP is an Intermediate filament protein specifically expressed in astrocytes in the CNS, and it serves as a sensitive and specific indicator of CNS plasticity in neurotoxic conditions [35], [36]. Ina is another intermediate filament protein expressed only in neurons, and can cause CNS plasticity by facilitating axonal neurite elongation [37]. These data are consistent with previous reports showing that morphine administration impact GFAP expression in certain brain areas [38], and support previously published evidence showing that morphine tolerance is actually a disorder of neuroplasticity [39], [40].

We also identified several chaperone proteins in this study. Chaperones play a key role in preventing their target proteins from misfolding and aggregating into nonfunctional structures; hence, they ensure proper activity of the target proteins [41]. The phosphorylation level of an ER-specific chaperone, Hspa5 (also called GRP78), was significantly altered in the spinal cord after development of morphine tolerance [42], [43]. Hspa5 is a major ER chaperone controlling the protein quality in the ER and protecting cells from ER stress, which is characterized by accumulation of misfolded proteins in ER [42], [43]. Various stresses can upregulate the expression of Hspa5, and the overexpressed Hspa5 within and outside the ER to play a critical role in cell viability [44]–[46]. For example, Hspa5 protects neurons and astrocytes against mitochondria dysfunction and stress-induced apoptosis [44], [46]. The effect of morphine on Hspa5 is consistent with previous reports demonstrating that another ER chaperone, BiP, also plays a pathophysiologic role in the development of morphine tolerance [47].

Two of the identified chaperones belong to the 14-3-3 protein family [48], [49]. 14-3-3 proteins are able to bind to the phosphorylated motifs of their partner proteins to affect protein activities [29]. Through functional modulation of the binding partners, 14-3-3 proteins are involved in multiple processes running in the cell, including metabolism, apoptosis, cell cycle and gene transcription [50], [51]. It has been known that posttranslational modifications playing important roles in regulation of 14-3-3 activity, and this regulation plays an important role in cytoskeleton reorganization of intermediate filaments in neurons [52]. Especially, 14-3-3 proteins modulate the sensitivity and trafficking of opioid and NMDA receptors [53], [54], both of which are known to be involved in the development of morphine tolerance [39]. Thus, the dysregulated phosphorylation of 14-3-3 proteins seen in this study might contribute to the pathophysiology of morphine tolerance through opioid and/or NMDA receptors.

Like chaperones, scaffold proteins are a type of regulatory protein that modulates cellular processes through interaction with multiple partner proteins. In our study, we identified Phb, a scaffold protein originally found in mitochondria [55]. Within mitochondria, Phb is able to maintain mitochondrial integrity and suppress free radical production [56]. However, Phb also has functions outside of mitochondria, such as controlling proliferation, apoptosis, and transcription [57]. In addition, Phb is neuroprotective against stress-induced neuronal death [56], and plays a role in modulating PI3K/Akt and Ras/MAPK/ERK signal transduction [30]. The altered phosphorylation state of Phb in morphine-treated rats suggests that morphine influences some of the known functions of Phb in the spinal cord.

In addition to the structural and regulatory proteins described above, our results also demonstrated that local injection of morphine into spinal cord disturbed the phosphorylation of two glycolysis metabolic enzymes (Eno2 and Aldoc) in the injected spinal area. This finding is consistent with previous reports showing that systematic treatment of morphine dysregulates metabolic enzymes and alters the functional state of carbohydrate metabolism in both the CNS and peripheral organs [58]–[60]. Interestingly, Eno2 is an enzyme highly expressed in CNS, and has neurotrophic and neuroprotective effects on a broad spectrum of CNS neurons [61], [62]. It can be inferred that the neuronal effects may also be associated with the development of morphine tolerance.

In conclusion, our proteomics data showed that repeated intrathecal injection of morphine dysregulated the phosphorylation of 10 proteins in the spinal cord. Bioinformatics analysis revealed five functional networks of proteins that are affected and known to be involved in cytoskeletal reorganization, neuroplasticity, protein folding and modulation, signal transduction, and biomolecular metabolism (Fig. 5A). These proteins are known to be expressed in different subcellular organells, associating with neuroplasticity, ER stress, mitochondrial dsyfunction, receptor trafficking and neuroprotection (Fig. 5B). Our data may shed light on the multiple phosphoproteome mechanisms that underlie the development of morphine tolerance.
